# The *in vivo* properties of STX243: a potent angiogenesis inhibitor in breast cancer

**DOI:** 10.1038/sj.bjc.6604707

**Published:** 2008-10-07

**Authors:** M F C Parsons, P A Foster, S K Chander, R Jhalli, S P Newman, M P Leese, B V L Potter, A Purohit, M J Reed

**Affiliations:** 1 Endocrinology and Metabolic Medicine and Sterix Ltd., Faculty of Medicine, Imperial College London, St Mary's Hospital, London W2 1NY, UK; 2 Medicinal Chemistry, Department of Pharmacy and Pharmacology and Sterix Ltd., University of Bath, Bath BA2 7AY, UK

**Keywords:** angiogenesis, breast cancer, pharmacokinetics, *bis-*sulphamates, 2-methoxyoestradiol

## Abstract

The steroidal-based drug 2-ethyloestradiol-3,17-*O*,*O*-*bis*-sulphamate (STX243) has been developed as a potent antiangiogenic and antitumour compound. The objective of this study was to ascertain whether STX243 is more active *in vivo* than the clinically relevant drug 2-methoxyoestradiol (2-MeOE2) and the structurally similar compound 2-MeOE2-3,17-*O*,*O*-*bis*-sulphamate (STX140). The tumour growth inhibition efficacy, antiangiogenic potential and pharmacokinetics of STX243 were examined using four *in vivo* models. Both STX243 and STX140 were capable of retarding the growth of MDA-MB-231 xenograft tumours (72 and 63%, respectively), whereas no inhibition was observed for animals treated with 2-MeOE2. Further tumour inhibition studies showed that STX243 was also active against MCF-7 paclitaxel-resistant tumours. Using a Matrigel plug-based model, *in vivo* angiogenesis was restricted with STX243 and STX140 (50 and 72%, respectively, using a 10 mg kg^−1^ oral dose), thereby showing the antiangiogenic activity of both compounds. The pharmacokinetics of STX243 were examined at two different doses using adult female rats. The compound was orally bioavailable (31% after a single 10 mg kg^−1^ dose) and resistant to metabolism. These results show that STX243 is a potent *in vivo* drug and could be clinically effective at treating a number of oncological conditions.

The hypothesis that tumour development is dependent on angiogenesis was originally proposed by Folkman (1971). This discovery has lead to the development of numerous antiangiogenic inhibitors for treating a variety of diseases, including lung and colon cancer (Eskens, 2004; Folkman, 2006).

An angiogenesis inhibitor that is currently under clinical investigation is the steroidal-based compound 2-methoxyoestradiol (2-MeOE2; Figure 1.1) (Kerbel and Folkman, 2002). The biological properties of 2-MeOE2 were first investigated by Fotsis *et al* (1994). *In vitro* studies have shown that 2-MeOE2 has antiproliferative and proapoptotic effects on endothelial cells (Fotsis *et al*, 1994; Tsukamoto *et al*, 1998). However, high concentrations of drug are required *in vivo* to inhibit tumour growth, and some studies have shown that 2-MeOE2 is ineffective at suppressing the growth of certain tumours (Fotsis *et al*, 1994; Ryschich *et al*, 2003; Ireson *et al*, 2004). In addition, clinical trials have shown that 2-MeOE2 needs to be administered at doses ranging from 200 to 1200 mg to achieve a therapeutic effect in prostate and breast cancer patients (Lakhani *et al*, 2003; Sweeney *et al*, 2005; Dahut *et al*, 2006). The pharmacological properties of 2-MeOE2 may be responsible for the relatively high dose of drug required in these studies. A recent study has shown that 2-MeOE2 was rapidly converted to inactive 2-methoxyoestrone in cells that expressed high levels of 17*β*-hydroxysteroid dehydrogenase type 2 (Newman *et al*, 2006), an enzyme that is expressed at high concentrations in the epithelial cells of the gastrointestinal tract (Mustonen *et al*, 1998). This finding may explain why an extensive metabolism of 2-MeOE2 to 2-methoxyoestrone was observed in a phase I study of 15 patients with metastatic breast cancer (Lakhani *et al*, 2003). 2-Methoxyoestradiol shows very poor oral bioavailability; no detectable concentrations of the drug were found in the plasma of female Wistar rats after they had received a single 10 mg kg^−1^ oral dose (Ireson *et al*, 2004). In support of this finding, a phase I trial was terminated because of the very low levels of 2-MeOE2 detected in the patients’ plasma (Dahut *et al*, 2006). New steroidal-based compounds are currently being developed with the objective of increasing potency and oral bioavailability. Two of these compounds currently under preclinical investigation are 2-MeOE2-3,17-*O*,*O*-*bis*-sulphamate (STX140; Figure 1.2) and 2-ethyloestradiol-3,17-*O*,*O*-*bis*-sulphamate (STX243; Figure 1.3).

Earlier investigations have shown that STX140 is a potent anticancer compound both *in vitro* and *in vivo*. STX140 was found to inhibit human umbilical vein endothelial cell (HUVEC) proliferation, a model widely used for studying angiogenesis. This study showed that STX140 was 60-fold more effective than 2-MeOE2 at inhibiting proliferation (Newman *et al*, 2004). Earlier studies have also shown that STX140 initiates apoptosis, possibly by phosphorylating BCL-2 and activating caspases 3 and 9 (Day *et al*, 2003; Wood *et al*, 2004). *In vitro* STX140 has been shown to inhibit cell proliferation, including preventing the growth of cells that are resistant to conventional chemotherapeutic agents (Suzuki *et al*, 2003; Raobaikady *et al*, 2005). *In vivo* investigations have shown that STX140 is orally effective at inhibiting the growth of both oestrogen-receptor-positive (ER+) and oestrogen-receptor-negative (ER−) tumours (Ireson *et al*, 2004; Utsumi *et al*, 2005). In contrast, when 2-MeOE2 is administered at the same dose, no inhibition of tumour growth was observed (Ireson *et al*, 2004). STX140 overcomes the oral bioavailability problems encountered with 2-MeOE2. When female Wistar rats received a single 10 mg kg^−1^ oral dose, significant concentrations of STX140 were found in plasma after 24 h. In the same study, 85% oral bioavailability was attained and no major metabolic products of STX140 were identified (Ireson *et al*, 2004).

Preliminary investigations have been performed to examine the *in vitro* properties of STX243. Studies conducted with a range of cell lines have shown that STX243 is equipotent to STX140 at inhibiting cell proliferation (Suzuki *et al*, 2003; Raobaikady *et al*, 2005). Novel experiments recently conducted by our group have shown that STX243, and also STX140, induce G_2_/M arrest and apoptosis in cells recovered from xenograft tumours (Foster *et al*, 2008).

In this study, investigations were undertaken to compare the *in vivo* efficacy of STX243 with STX140 and 2-MeOE2. Tumour growth inhibition was assessed in two breast cancer animal models. MDA-MB-231 xenograft tumours were used to compare the *in vivo* efficacy of all three compounds, and a tumour resistance study was conducted to investigate the ability of STX243 to inhibit the growth of paclitaxel-resistant MCF-7dox40 xenograft tumours. The antiangiogenic potential of STX243 was also evaluated using an established Matrigel plug-based methodology (Passaniti *et al*, 1992; Prewett *et al*, 1999; Chander *et al*, 2007). To ascertain whether the biological properties of STX243 could overcome the oral bioavailability problems encountered with 2-MeOE2, the pharmacokinetics of STX243 were examined in adult female Wistar rats. The pharmacokinetics of STX140 and 2-MeOE2 have been evaluated earlier by our group (Ireson *et al*, 2004).

## Materials and methods

### Compound synthesis

2-Methoxyoestradiol, STX140, STX243 and its putative metabolite 2-ethyloestradiol-17-*O*-sulphamate (STX1813) were synthesised as described earlier (Leese *et al*, 2005, 2006). Spectroscopic and analytical data were obtained in accordance with each compound’s structure. The purity of the compounds was confirmed through the use of high-performance liquid chromatography (HPLC). CLogP (Leo, 2003) was calculated using ChemOffice 2008, Cambridgesoft, Cambridge, MA, USA.

### Angiogenesis assay

The effects of the compounds on *in vitro* vessel formation were assessed using an angiogenesis kit (TCS Cellworks, Claydon, UK). For this assay, HUVECs (obtained from TCS Cellworks) were cultured in a 24-well plate within a matrix of human diploid fibroblasts of dermal origin in an optimised medium (TCS Cellworks) supplemented with 2 ng ml^−1^ vascular endothelial growth factor (VEGF). The cocultured cells were incubated throughout the experiment at 37°C under 5% CO_2_ in a humidified incubator. On day 1, the culture medium was removed and replaced with medium containing the compounds under investigation. The vehicle used for the dilution of compound was tetrahydrofuran (THF). The final concentration of THF in the medium was 0.001 (100 nM dose) to 0.00025% (25 nM dose). On days 4, 7 and 9, the medium was replaced with fresh medium containing the compounds. Each compound was tested in triplicate. On day 11, the cells were washed (phosphate-buffered saline) and 70% ethanol (1 ml) was added to each well for 30 min to fix the cells. After fixation, the cells were washed with blocking buffer (1 ml, phosphate-buffered saline+1% bovine serum albumin) and stained for CD31 according to the manufacturer's instructions (TCS Cellworks).

The extent of vessel formation was then quantified using a variation of an earlier described technique (Newman *et al*, 2004). Briefly, using a high-resolution transmissive scanner (ScanMaker 9800, Microtek, Willich, Germany), each well was scanned and saved as a tagged image format (TIF) file in Photoshop (Adobe, San Jose, CA, USA). The image was then converted to a black and white image using the photocopy filter in Photoshop ( × 2, 10 detail and 25 darkness) and saved as an uncompressed TIF file. The files were transferred to the AngioSys software (TCS Cellworks), all background and non-tubule-like structures were removed using erode ( × 1) and clean (100 pixels) functions, and the number of pixels representing vessels was counted. This technique was validated (data not shown) against the quantification techniques as described earlier (Newman *et al*, 2004).

### *In vivo* experiments

All animal experiments were conducted in accordance with the United Kingdom Co-ordinating Committee on Cancer Research (UKCCCR) guidelines for the Welfare of Animals in Experimental Neoplasia (Workman *et al*, 1998) and approved by the Imperial College Ethical Review Committee. The animals were maintained in positive pressure isolators with 12 h light and dark cycles. Food and water were available to the animals *ad libitum*. The rats received expanded RM1 polylined diet, whereas the mice received irradiated CRM pellets (Special Diet Services, Witham, Essex, UK).

### Tumour growth inhibition models

The tumour growth inhibition efficacy of STX243 was investigated in two different breast cancer mice models. In both models the animals were 6 weeks of age at the start of study.

An MDA-MB-231 (ER−) cell-based model was used to compare the *in vivo* properties of STX243, STX140 and 2-MeOE2. The MDA-MB-231 cells were kindly donated by Dr PG Kasprzyk (IPSEN-Biomeasure, Milford, MA, USA). In this study, 2 × 10^6^ cells were injected subcutaneously into the flank of female MF-1 nu/nu mice (Harlan, Bicester, Oxon, UK). Oral administration of vehicle (10% THF, 90% PG), STX140 (20 mg kg^−1^), STX243 (40 mg kg^−1^) and 2-MeOE2 (40 and 75 mg kg^−1^) was initiated when the tumours reached approximately 100 mm^3^. An earlier study had shown the efficacy of STX140 at this dose (Ireson *et al*, 2004). Furthermore, the maximum-tolerated dose from a single oral administration is 150 mg kg^−1^ for STX140 and 300 mg kg^−1^ for STX243 (Foster *et al*, 2008). The animals were dosed daily for 21 days. Each treatment group comprised five animals, except for the vehicle-only group with seven animals. The weight of the animals was recorded at regular intervals throughout the study. Tumour measurements were taken every week using electronic calipers and the volume of the tumours was determined using the formula: *length* × *width*^2^/2 (*l* × *w*^2^/2).

The potential of STX243 to inhibit the growth of paclitaxel-resistant tumours was investigated using two MCF-7 (ER+) cell lines. The MCF-7 wild-type (MCF-7wt) cells were purchased from the American Type Culture Collection (ATCC; LGC Promochem, Teddington, Middlesex, UK) and the MCF-7 doxorubicin-resistant (MCF-7dox40) cells were kindly donated by Dr GL Scheffer (Department of Pathology, Free University Hospital, Amsterdam, Netherlands). MCF-7dox40 is a paclitaxel-resistant cell line because of its overexpression of the multidrug resistance protein, P-glycoprotein (Scheffer *et al*, 2000). In this study, 5 × 10^6^ MCF-7wt and MCF-7dox40 cells in Matrigel were injected subcutaneously into the right and left flank, respectively, of female MF-1 nu/nu mice. When the tumours reached 70–100 mm^3^ in volume, the animals were dosed either orally with STX243 or vehicle, or intravenously with paclitaxel. The orally dosed animals received daily administrations of vehicle (10% THF, 90% PG) or STX243 (40 mg kg^−1^), whereas the paclitaxel (15 mg kg^−1^) animals received weekly intravenous injections of the drug dissolved in 0.9% (w/v) NaCl (saline). The animals were dosed for a total of 28 days with five animals in each treatment group. The weight of the animals was recorded at regular intervals and the volume of the tumours was measured on a weekly basis using the formula described earlier.

### *In vivo* angiogenesis assay

The antiangiogenic potential of STX243 was assessed using a modified Matrigel plug assay as described earlier (Passaniti *et al*, 1992; Prewett *et al*, 1999; Chander *et al*, 2007). The female C57BL/6J mice used in this study were obtained from Harlan (Bicester). The mice were anaesthetised, placed on a heated pad (37°C) and 0.5 ml of ice-cold Matrigel supplemented with 500 ng of basic fibroblast growth factor (bFGF) was injected subcutaneously into the flanks of each mouse. Control mice received Matrigel without bFGF. The mice were subsequently weighed before returning the mice to their isolator. After 24 h of Matrigel injection, the mice were placed into the following treatment groups: control, bFGF-only, bFGF plus STX140 (10 mg kg^−1^, orally) and bFGF plus STX243 (10, 20 or 40 mg kg^−1^, orally). Control and bFGF-only animals were dosed orally with vehicle (10% THF, 90% PG). The animals were dosed every day for four days with five animals per treatment group. Quantification of the blood vessels within the Matrigel plugs was facilitated through the intravenous injection of 100 *μ*l of 0.25 mg ml^−1^ FITC-dextran (250 000 molecular weight) seven days after the Matrigel injection. FITC-dextran injection allows for visualisation of the blood vessels that has been recently formed in the matrigel plugs. The animals were killed 20 min after the injection of the FITC-dextran. The mice were subsequently weighed and the Matrigel plugs were removed. The Matrigel plugs were photographed and subsequently dissolved in 1 ml Dispase reagent for 16 h at 37°C. The resulting mixture was centrifuged at 15 700 **g** (20°C) for 30 s. The fluorescence of the supernatant was measured using a FLUOstar OPTIMA fluorimeter (BMG Labtech Ltd., Aylesbury, Bucks, UK). The excitation and emission wavelengths used were 480 and 520 nm, respectively. The fluorescence of the samples was quantitated using a standard curve of FITC-dextran (0.4–25 *μ*g ml^−1^).

### Pharmacokinetic studies

The pharmacokinetic properties of STX243 were investigated using female Wistar rats (Charles River, Margate, Kent, UK). The rats were weighed and STX243 was administered either intravenously or orally at two different doses (10 or 40 mg kg^−1^). Control animals only received vehicle (10% THF, 90% PG). At specified time intervals after dosing, blood was removed by cardiac puncture under terminal anaesthesia (isoflurane), the rats were culled through cervical dislocation and the weight of each rat was recorded. The time points used in this study were as follows: 5 and 30 min; 1, 2, 3, 4, 8 and 24 h, with three animals per time point. Plasma was separated from blood cells by centrifugation (1900 **g**, 4°C, 10 min) and stored at −80°C until it was required for analysis. After the samples had defrosted, the plasma was divided into 0.5 ml samples and progesterone (0.1 mM) was added to the sample to act as an internal standard. Through the use of solid phase extraction (SPE), STX243 was extracted from the samples under vacuum using Oasis HLB Extraction Cartridges (Waters, Elstree, Herts, UK). The column was prepped with methyl-tert-butyl ether, methanol and water, before passing the sample down the column. The column was subsequently washed with methanol/water (1 : 19, v/v). Elution of the sample was achieved using methanol/methyl-tert-butyl ether (1 : 9, v/v). The eluant was evaporated to dryness under a stream of air at 40°C. The residues were analysed immediately by HPLC. The extraction efficiency of STX243 from plasma was found to be 54±2% (mean±s.e.m.).

### HPLC analysis

Sample analysis was performed using a reverse phase HPLC method. The residues were reconstituted in mobile phase and 100 *μ*l of each sample was injected onto an Agilent 1100 ChemStation HPLC system (Wokingham, Berks, UK). Mobile phase consisted of 65% methanol in 0.02 M ammonium sulphate (pH 6.5). STX243, STX1813 and progesterone were separated from endogenous plasma components using a Gemini 5 *μ* C6-Phenyl 110 column (250 × 3.0 mm; Phenomenex, Macclesfield, Cheshire, UK) and a flow rate of 0.425 ml min^−1^. The column temperature was set at 25°C and the samples were analysed using a photodiode array detector set to 202 or 220 nm. Two different wavelengths were used because preliminary investigations showed that the lower wavelength was required to analyse the samples that contained low levels of STX243, and accurate quantification of high concentrations was only achieved when a wavelength of 220 nm was employed. Quantification was achieved by spiking plasma with known amounts of STX243 and the internal standard, progesterone, followed by extracting the samples with SPE as outlined earlier. The concentration of STX243 in the plasma samples was quantified using linear calibration curves in the range of 6–100 000 ng ml^−1^. The limits of detection (LOD) and limits of quantification were 1.8 and 6 ng ml^−1^, respectively. The intra-day and inter-day coefficients of variation were 2.9 and 3.8%, respectively.

### Pharmacokinetic analysis

The pharmacokinetic parameters were calculated using WinNonlin software (Pharsight, Mountain View, CA, USA). A non-compartmental model was used to evaluate the plasma concentration data attained after oral administration. A two-compartmental approach gave the best approximation to the intravenous data attained. The pharmacokinetic parameters were attained by initially calculating the area under the curve (AUC), the area under the first moment *vs* time curve (AUMC) and the distribution and elimination constants (*α* and *β*). From these results, it was possible to calculate the following parameters: distribution half-life (*t*_1/2_*α*)=LN(2)/*α*, elimination half-life (*t*_1/2_*β*)=LN(2)/*β*, mean residence time (MRT)=AUMC/AUC, total body clearance (Cl)=dose/AUC, volume of distribution (V_D_)=Cl^*^MRT and bioavailability (% F)=(AUC_p.o._/AUC_i.v._) × 100.

### Statistics

All *in vitro* experiments were carried out in triplicate. All errors shown are the mean±s.d. Student's *t*-test was used to assess the significance of the differences in cell proliferation *in vitro*. For xenograft data, one-way analysis of variance followed by a Bonferroni's multiple comparison test was performed to determine the statistical significance on most data sets. When only two groups are compared, Student's *t*-test was applied. All values are represented as the mean±s.d. Data generated in these studies were normally distributed as assessed by the method of Kolmogorov and Smirnov. The type-1 error is 0.05. Statistics were calculated using Prism 3 for Mac (GraphPad Software Inc., San Diego, CA, USA).

## Results

### *In vitro* angiogenesis

**Figure 2:**
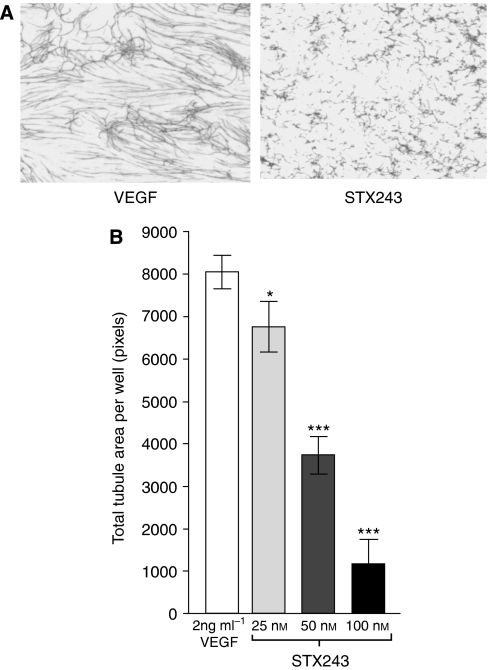
Inhibition of angiogenesis by STX243 *in vitro.* STX243 disrupts the ability of endothelial cells to form vessel-like structures. The non-computer enhanced high-resolution scans of each well show the staining of the CD31-positive endothelial cells, which form vessel-like structures (**A**). A dose-dependent effect of STX243 on blood vessel formation is observed. All columns represent cells grown in VEGF supplemented media (**B**). Data represent mean±s.d.; *n*=3; ^*^P<0.05 and ^***^P<0.001 significance compared with the control group.

In this study, a coculture model was used, in which endothelial cells are cocultured with fibroblasts in an optimised medium from TCS Cellworks. The pro-angiogenic factor VEGF (2 ng ml^−1^) was used to further stimulate vessel formation in this assay, and the capacity for STX243 to inhibit the VEGF-stimulated angiogenesis was assessed. The representative high-resolution scans of the wells clearly show that 100 nM STX243-inhibited VEGF-stimulated vessel formation (Figure 2A). Quantification of the scans, using an earlier validated method (Newman *et al*, 2004), shows that STX243 at 100 nM completely blocked all tubule formation (Figure 2B). At 50 nM, STX243 still causes a significant inhibition of vessel formation. However, at 25 nM, the effects of STX243 are less pronounced, resulting in only a small inhibition.

### *In vivo* tumour efficacy

**Figure 3:**
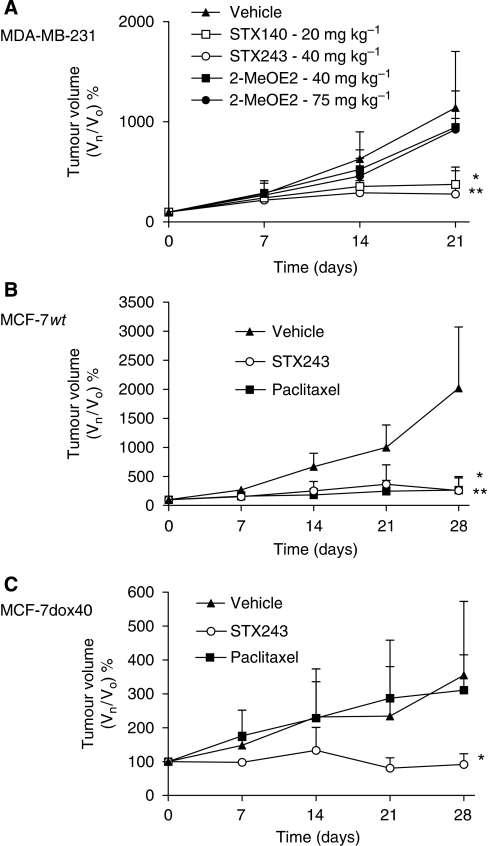
Inhibition of tumour growth in MF-1 nude mice xenograft models. In the MDA-MB-231 study (**A**) the animals were randomised into five treatment groups when the tumours reached the required volume. These groups were as follows: vehicle-only, STX140 (20 mg kg^−1^, orally), STX243 (40 mg kg^−1^, orally) and 2-MeOE2 (40 and 75 mg kg^−1^, orally). All of the compounds were administered on a daily basis. In the MCF-7wt (**B**) and MCF-7dox40 (**C**) investigations, the mice were randomised to receive vehicle-only (daily), STX243 (40 mg kg^−1^, orally daily) and paclitaxel (15 mg kg^−1^, intravenous weekly). Data represent mean±s.d., *n*=5 for all groups, except for the vehicle only group for Figure 3A when *n*=7. Each drug treatment was compared with the control group for statistical significance: *P<0.05 and **P<0.01.

In the MDA-MB-231 study, significant tumour growth inhibition was observed in the mice that were dosed daily with STX243 (40 mg kg^−1^, orally) and STX140 (20 mg kg^−1^, orally) (Figure 3A). Compared with the vehicle-only animals, tumour growth inhibition of 72±13% (*P*<0.01) and 63±7% (*P*<0.05) was attained with STX243 and STX140, respectively, after 21 days of drug administration. No significant tumour inhibition was observed with either dose of 2-MeOE2 (40 or 75 mg kg^−1^, orally daily) used in this investigation.

MCF-7wt and paclitaxel-resistant (MCF-7dox40) xenograft tumours were used in the *in vivo* resistance study. Both STX243 and paclitaxel significantly inhibited the growth of the MCF-7wt tumours (*P*<0.01 and *P*<0.05, respectively; Figure 3B). Compared with the vehicle-only animals, the percentage of tumour growth inhibition for STX243 and paclitaxel was 87±5% and 87±6%, respectively. In contrast, only STX243 was capable of significantly inhibiting the growth of the MCF-7dox40 tumours (*P*<0.05; Figure 3C). In comparison with the vehicle-only animals, growth inhibition of 74±4% was observed with STX243 at the end of the 28-day dosing period.

### *In vivo* angiogenesis assay

**Figure 4:**
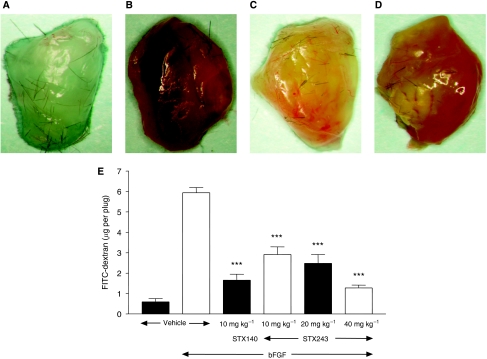
The inhibition of angiogenesis in C57BL/6J mice Matrigel plug-based model. The Matrigel plugs were photographed to show the extent of vascularisation after the mice had received the following treatments: control (**A**), bFGF-only (**B**), bFGF and STX140 (10 mg kg^−1^ orally) (**C**) and bFGF and STX243 (10 mg kg^−1^ orally) (**D**). The plugs were subsequently dissolved with Dispase and the fluorescence of the FITC-dextran was used to quantify the extent of angiogenesis (**E**). Data represent mean±s.d., *n*=5. Each drug treatment was compared with the bFGF-only group for statistical significance: ^***^P<0.001.

*In vivo* angiogenesis in Matrigel plugs without bFGF was not observed (Figure 4A). The addition of bFGF to Matrigel stimulated angiogenesis and caused a >10-fold increase in fluorescence of the plugs (Figure 4B and E). This growth of new blood vessels was significantly inhibited by administering either STX243 or STX140 to the animals (*P*<0.001 for all of the doses investigated) (Figure 4C, D and E). When compared with the animals that received bFGF-only, the level of angiogenesis was reduced by 50±6% using 10 mg kg^−1^ of STX243 and 72±5% using 10 mg kg^−1^ of STX140. When the dose of STX243 was increased, the level of fluorescence associated with the Matrigel plugs decreased.

### Pharmacokinetics of STX243

**Figure 5:**
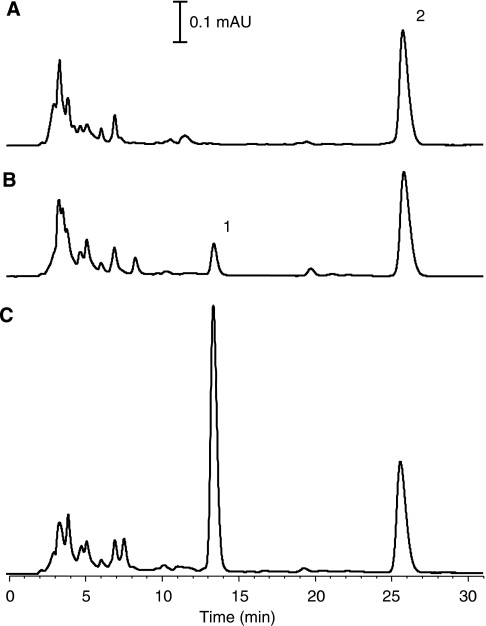
HPLC chromatograms from the STX243 pharmacokinetic experiments. STX243 was administered either intravenously or orally at two different doses (10 or 40 mg kg^−1^) to female Wistar rats. Control animals received vehicle-only. STX243 was extracted from the plasma using solid phase extraction and the extracts were analysed using HPLC. The chromatograms shown are: control (**A**), STX243 (40 mg kg^−1^, orally) (**B**) and STX243 (40 mg kg^−1^, intravenously) (**C**). In the chromatograms shown, the rats were culled 3 h after dosing with STX243. Peaks 1 and 2 correspond respectively to STX243 and the internal standard (progesterone).

The pharmacokinetic parameters of STX243 were assessed in adult female Wistar rats. Earlier we have shown the pharmacokinetics of STX140 (Ireson *et al*, 2004). Figure 5 shows typical HPLC traces that were obtained after the animals were dosed either intravenously or orally with 40 mg kg^−1^ of STX243. Using the HPLC method described earlier, STX243 was separated from the putative metabolite STX1813 and the internal standard progesterone. The retention times for the three compounds were 13.4, 16.2 and 25.8 min for STX243, STX1813 (data not shown) and progesterone, respectively.

**Figure 6:**
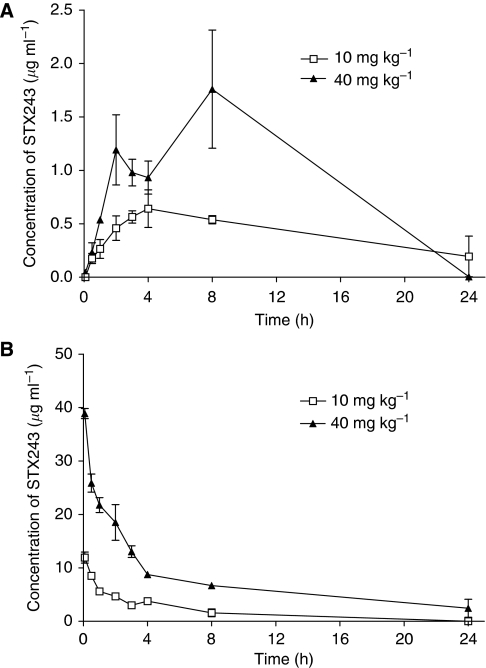
Concentration of STX243 in rat plasma after a single oral administration (**A**) or a single intravenous injection of STX243 (**B**). Animals were dosed with either 10 or 40 mg kg^−1^ STX243. Plasma samples were evaluated according to the HPLC analysis procedure described in the Materials and Methods section. Data represent mean±s.d., *n*=3.

STX243 was detected at all of the time points studied apart from the initial 5-min time point after the animals were dosed orally with 10 mg kg^−1^ of STX243 (Figure 6). The highest concentrations of STX243 were found in the plasma 4 and 8 h after the animals had been orally dosed with 10 or 40 mg kg^−1^ of STX243 respectively. The bioavailability of STX243 increased from 18 to 31% when the administered dose was lowered from 40 to 10 mg kg^−1^ (Table 1). Similar distribution (*t*_1/2_*α*) and elimination (*t*_1/2_*β*) half-life values were attained after intravenous administration regardless of the dose of STX243 administered. The calculated elimination half-life of STX243 was 1.88 h after 40 mg kg^−1^ oral administration. However, an increased elimination half-life of 11.40 h was attained when the mice were dosed orally with 10 mg kg^−1^. The increased value was primarily because of the high concentration of STX243 detected in the plasma 24 h after dosing. High clearance and volume of distribution values were observed under all dosing conditions. The values observed were particularly apparent when STX243 was administered orally, with clearance values above 700 ml kg^−1^ h^−1^ and volumes of distribution above 12 l kg^−1^ for both doses under investigation. No metabolism of STX243 to STX1813 was detected in any of the plasma samples.

None of the animals lost a significant amount of weight in any of the investigations conducted.

## Discussion

The steroidal-based entity STX243 has been developed as a potentially active antiangiogenic and antitumour drug. The objectives of this investigation were to assess the *in vivo* properties of STX243 and compare the biological properties of this drug with two structurally similar compounds STX140 and 2-MeOE2. Tumour growth inhibition was examined in two breast cancer models and the antiangiogenic potential of STX243 was evaluated using a Matrigel plug-based assay. Preclinical studies have shown that the addition of sulphamate groups to steroidal-based compounds can increase their bioavailability, as showed by earlier investigations that compared the pharmacokinetics of 2-MeOE2 with STX140 in female Wistar rats (Ireson *et al*, 2004). To compare the biological properties of all three compounds, the same animal model was used in this study to examine the pharmacokinetics of STX243.

STX243 is a potent orally active compound, as highlighted by the tumour growth inhibition studies. The results of the MDA-MB-231 xenograft study showed that there was no significant difference between the level of tumour growth inhibition obtained with 40 mg kg^−1^ of STX243 and 20 mg kg^−1^ of STX140 (*P*>0.05). In contrast to the results with STX140 and STX243, 2-MeOE2 did not inhibit the growth of the tumours at either dose used (40 or 75 mg kg^−1^) in this investigation. This finding is in agreement with numerous *in vitro* and *in vivo* investigations. Earlier cell proliferation studies have shown that STX243 and STX140 are considerably more potent than 2-MeOE2, with some investigations highlighting a 10-fold difference in activity (Day *et al*, 2003; Wood *et al*, 2004; Leese *et al*, 2005). *In vivo* STX140 has showed the capability to restrict tumour growth, whereas 2-MeOE2 has been shown to be comparatively ineffective (Ryschich *et al*, 2003; Ireson *et al*, 2004; Utsumi *et al*, 2005).

The growth of MCF-7 xenograft tumours was also inhibited by STX243, thereby proving that the steroidal-based compound is active against both ER+ and ER− tumours. In this study, both STX243 and paclitaxel were capable of inhibiting the growth of the MCF-7wt tumours. However, the growth of the MCF-7dox40 tumours was inhibited only by STX243. This finding indicates that STX243 is not a substrate for the multidrug resistance protein, P-glycoprotein, which is overexpressed in this cell line (Scheffer *et al*, 2000). The results obtained here with paclitaxel are supported by earlier *in vivo* investigations that have shown that the taxane is ineffective at inhibiting the growth of breast carcinoma tumours that overexpress P-glycoprotein (Newman *et al*, 2000; Loganzo *et al*, 2003). Recent studies have shown that STX140 is also capable of inhibiting the growth of MCF-7dox40 tumours (Newman *et al*, 2008).

The results of the Matrigel plug-based assay clearly show the antiangiogenic potential of STX243. The steroidal-based compound significantly inhibited the angiogenesis of the bFGF-stimulated Matrigel plugs. Comparable levels of fluorescence were found in the plugs from the animals treated with either STX243 or STX140. However, in agreement with the tumour growth inhibition studies, a higher dose of STX243 was required to obtain the same antiangiogenic effect. Three doses of STX243 were used in this study; as the dose increased there was a corresponding decrease in the extent of angiogenesis. Earlier experiments in our laboratory have shown that STX140 is more efficacious than 2-MeOE2 in reducing the level of angiogenesis in Matrigel plugs. Angiogenesis inhibition was only observed when the mice were dosed orally with 50 mg kg^−1^ of 2-MeOE2, whereas no significant inhibition was observed using a 10 mg kg^−1^ dose of the drug (Chander *et al*, 2007). Judging from the *in vitro* angiogenesis kit results, it is likely that STX243 disrupts new vessel formation *in vivo*, and therefore results in a tumour growth reduction.

The pharmacokinetics of STX140 and 2-MeOE2 have been analysed earlier using an identical model as described here. Both drugs were administered to the animals orally and intravenously using a dose of 10 mg kg^−1^. The maximum concentrations of STX140 found in the plasma after oral and intravenous dosing were 3.9 and 6.6 *μ*g ml^−1^, respectively, and an average oral bioavailability of 85% was observed. In contrast, 2-MeOE2 was rapidly cleared from the plasma. Using HPLC, no 2-MeOE2 was detected in the plasma after oral administration and 1 h after intravenous dosing the concentration of 2-MeOE2 in the plasma was below the LOD (Ireson *et al*, 2004).

The results obtained in this study with STX243 show that the compound can be easily detected in plasma up to 24 h after intravenous or oral administration, using doses of 10 or 40 mg kg^−1^. This finding indicates that STX243 is considerably more biologically available than 2-MeOE2. The bioavailability of STX243 was 18% when the drug was administered at a dose of 40 mg kg^−1^ and increased to 31% when the dose was lowered to 10 mg kg^−1^. Therefore, in this pharmacokinetic model, STX243 is less bioavailable than STX140. Despite the differences in oral bioavailability, a similar plasma profile was observed for both drugs. For example, the peak concentration of STX243 in plasma was achieved 4 h after oral administration, when a dose of 10 mg kg^−1^ was used. Similarly, the peak plasma concentration of STX140 was obtained 3 h after oral administration; the 4 h time point being omitted. For both drugs, a two-compartment model was found to be the best approximation to the intravenous data obtained. In addition, no significant metabolism of STX243 or STX140 was observed after intravenous or oral administration in either of the studies (Ireson *et al*, 2004).

The pharmacokinetics of STX243, STX140 and 2-MeOE2 may provide a possible explanation for the angiogenesis and tumour growth inhibition data attained. In the tumour growth inhibition studies, 2-MeOE2 was ineffective at retarding tumour growth at either dose used. This finding is not surprising, as 2-MeOE2 displays very limited oral bioavailability. Both STX243 and STX140 are capable of inhibiting tumour growth and restricting the level of angiogenesis following oral administration. However, to achieve the same therapeutic effect, a higher dose of STX243 than STX140 was required. This finding is supported by the pharmacokinetic results, which shows that STX140 has higher bioavailability than STX243 when both drugs are administered orally at the same dose. In addition, these studies indicate that STX243 is removed comparatively quickly from plasma, as shown by the higher half-life times generally observed for STX140 and the high clearance levels calculated for STX243 (Ireson *et al*, 2004).

The sole structural difference between STX140 and STX243 is the replacement of the oxygen atom of the 2-methoxy group with a methylene (CH_2_) group in STX243. Although this change is minimal, it is clearly sufficient to impact significantly on both *in vitro* antiproliferative activity and the observed, *in vivo*, antitumour activity. Given that the 2-methoxy and 2-ethyl groups of STX140 and STX243 are of near identical size, steric factors can be reasonably discounted. The effects of the respective C-2 substituents on the electron density of the A-ring are also unlikely to greatly influence the potential electrostatic *π*–*π* interactions between the aromatic ring and proximal residues in the site of action. It may be that the greater hydrophobic nature of the ethyl group of STX243 (with respect to the methoxy group of STX140) allows for enhanced interaction with the colchicine-binding site on tubulin, with which STX243 is presumed to interact, and thus delivers the observed increase in antiproliferative activity *in vitro* (Leese *et al*, 2005).

A key advantage of the 2-ethyloestradiol-derived STX243 over its 2-methoxy congener (STX140) is the facile and high yielding manner, in which it is synthesised. The key synthetic intermediate, 2-ethyloestradiol, is generated from oestradiol in six steps and greater than 50% overall yield with numerous crystallisable intermediates (thus allowing for removal of any potential oestrogenic impurity) and requiring only one chromatographic purification (Bubert *et al*, 2007). The synthesis of STX243 is thus highly efficient and well suited to the manufacture of large quantities of material, a significant consideration for candidate drugs.

In conclusion, the results presented here show the *in vivo* efficacy of the steroidal-based compound STX243. This study shows that STX243 has favourable pharmacokinetic properties and is capable of impeding the growth of both ER+ and ER− breast cancer tumours. The results of the Matrigel plug-based assay prove that STX243 is a potent angiogenesis inhibitor. Importantly, these studies have proved that the clinically relevant agent 2-MeOE2 is comparatively inactive *in vivo* at the doses used in this investigation. With proven preclinical efficacy, it is anticipated that STX243 will have considerable clinical potential in treating patients with various oncological conditions.

## Figures and Tables

**Figure 1:**
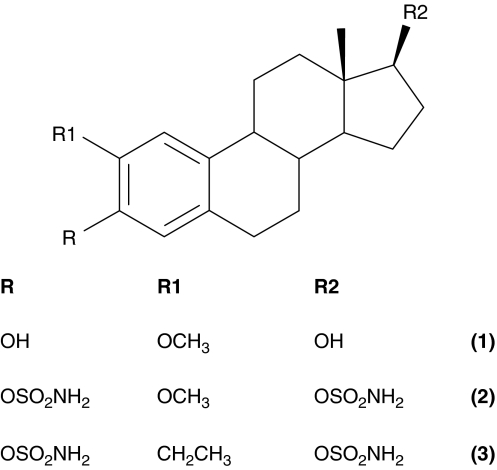
Chemical structures of 2-MeOE2 (1), STX140 (2) and STX243 (3).

**Table 1:** Summary of the pharmacokinetic data obtained after the oral or intravenous administration of STX243 at two separate doses (10 and 40 mg kg^−1^)

**PK parameter**	**Intravenous (40 mg kg^−1^)**	**Oral (40 mg kg^−1^)**	**Intravenous (10 mg kg^−1^)**	**Oral (10 mg kg^−1^)**
C_max_ (*μ*g ml^−1^)	44.85	1.76	13.14	0.64
*t*_1/2_*α* (h)	0.16	—	0.38	—
*t*_1/2_*β* (h)	3.18	1.88	4.78	11.40
AUC (h*μ*g ml^−1^)	126	23	42	13
AUMC (h^2^*μ*g ml^−1^)	558	157	267	219
MRT (h)	4.44	6.93	6.28	16.79
Cl (ml kg^−1^ h^−1^)	318	1764	235	766
V_D_ (l kg^−1^)	1.41	12.23	1.48	12.86
Bioavailability (% F)	—	18.0	—	30.7
